# An In Vitro Study of the Neurotoxic Effects of *N*-Benzylpiperazine: A Designer Drug of Abuse

**DOI:** 10.1007/s12640-016-9604-x

**Published:** 2016-02-09

**Authors:** Karolina Persona, Anna Polus, Joanna Góralska, Anna Gruca, Aldona Dembińska-Kieć, Wojciech Piekoszewski

**Affiliations:** Department of Analytical Chemistry, Faculty of Chemistry, Jagiellonian University in Krakow, Ingardena 3, 30-060 Kraków, Poland; Department of Clinical Biochemistry, Jagiellonian University in Krakow - Medical College, Kraków, Poland; School of Biomedicine, Far Eastern Federal University, Vladivostok, Russia

**Keywords:** New psychoactive substances, Benzylpiperazine, Apoptosis, Oxidative stress, Neurodegeneration

## Abstract

Recently, the number of new psychoactive substances has significantly increased. Despite the systematic introduction of prohibition in trade of medicinal products which mimic the effects of illegal drugs, the problem concerning this group of drugs is still important although knowledge about the mechanism of action of those types of substances is scarce. This study aimed to follow the neurotoxic effect of *N*-benzylpiperazine (BZP), the central nervous system psychostimulant, using the human cancer LN-18 cell model. The statistically significant elevation of LDH levels, increased mitochondrial membrane potential, decreased ATP and increased ROS production, increased levels of DNA damage marker (8-OHdG) and activation of caspases: -3 and -9 confirmed by Real-Time PCR imply the activation of mitochondrial proapoptotic pathways induced by BZP after 24 h incubation. This study is a novel, preliminary attempt to explain the toxicity of one of the most popular designer drug of abuse at the cellular level.

## Introduction

*N*-benzylpiperazine (BZP), also known as *A2*, *Legal X*, *Legal E*, or *Herbal Highs*, is a model compound from the group of new psychoactive compounds. The commercially available formulations of this substance advertised as a safe alternative to illegal drugs (Arbo et al. 2012) have very different trade names such as *Benny, Flying Angel, PEP, Twisted, Jax, Nemesis*, and *Red Eye Frog* (Gee et al. 2005). BZP is usually administrated orally in the form of tablets or capsules with different colors, shapes, and logo. The content of a new psychoactive compound in the formulations varies widely and depends mainly on the manufacturer. Average doses of BZP are estimated to be 50–200 mg (Sheridan et al. 2007) but there are known reports where a single dose was as high as 2000 mg (Wilkins et al. 2008). BZP is often combined with other piperazine derivatives: 1-(3-trifluoromethylphenyl) piperazine (TFMPP), 1-(3-chlorophenyl) piperazine (mCPP), and additives such as caffeine, guarana, or black pepper (Europol–EMCDDA 2007).

Studies carried out by the scientific team of Campbell et al. (1973) and Bye et al. (1973) have shown that the effects of BZP referred to as “positive” are tenfold weaker than the effects obtained after the administration of d-amphetamine. People who were either previously amphetamine dependent or without a history of substance abuse were unable to distinguish the effects of those substances such as psychomotor agitation or increased auditory sensations. The effects after taking 100 mg of BZP are maintained for about 6–8 h although the time might be dependent upon personal variation (EACD New Zealand 2004). Studies and observations presented in scientific papers have shown that BZP has a narrow margin of safety, even in the case of recreational use (Gee et al. 2008). Taking formulations containing BZP in their composition is thus linked to serious health risks and even death. The differences in the toxic effects of BZP are also contingent upon the occurrence of individual variability in the rate of biotransformation of BZP, particularly associated with the cytochrome P450 gene polymorphism and catechol-*O*-methyltransferase—involved in the metabolism of xenobiotics (Gee et al. 2005). The seizures observed in neurologically healthy patients which are a symptom of transient cerebral dysfunction as a result of the adverse effects of BZP may indicate the neurodegenerative properties of this xenobiotic (Gee et al. 2008). Another argument for the neurotoxicity of BZP may be the problems related to a working memory and the associated speed of thought processes observed in a patient after 5 months of substance poisoning (Gee et al. 2010).

Despite the comparatively long history of this designer drug of abuse dating back to the 1950s (Buck and Baltzly 1947), there is still little information on its mechanisms of action and the causes of its side effects. Taking into account the data about the toxic effects of BZP, the conduction of the research on the impact of this new psychoactive substance on human cell processes seems to be reasonable and useful for predicting the adverse effects of action (also derivatives of the compound or compounds with similar chemical structures), as well as for designing detoxication treatments.

Apoptosis is of great importance for the health and balance of the body. Extending properly programed cell death is of particular importance not only at the stage of embryogenesis and organogenesis, but also in proper functioning of the human body (Renehan et al. 2001). Both inhibited apoptosis and its excessive activation caused by cytotoxic compounds such as BZP might lead to serious complications such as autoimmune diseases (Eguchi 2001), neurodegenerative disorders (Mattson 2000; Ghavami et al. 2014), besides cancer (Wong 2011). Additionally, sympathomimetic compounds may lead to disruption of the energy state of cells or oxidative stress by excessive substrates or over-stimulation during the oxidative phosphorylation process (ROS) (Li et al. 2003; Lai et al. 2005; Leavesley et al. 2008; Quinzii and Hirano 2010). This paper is an attempt to clarify the effect of BZP on selected types of biological processes connected with the energy state of cells and oxidative stress, and apoptosis in the human glial cell line LN-18.

## Materials and Methods

### Cells Culture

The human glioblastoma cells (LN-18) originating from a patient with a right temporal lobe glioma (ATCC, CRL-2610) were cultured in ATCC-formulated Dulbecco’s Modified Eagle’s Medium, (ATCC Catalog No. 30-2002) with 50 µg/mL supplements of an antibiotics mixture of penicillin, streptomycin, and amphotericin B (Sigma-Aldrich) and 5 % Fetal Bovine Serum (Carlsbad) at an atmosphere of 95 % air and 5 % CO_2_ and temperature of 37.0 °C. In order to use them for experiments or when a cell’s confluence was close to 100 %, the cells were passaged to a new culture vessel (Corning Costar^®^) using a 0.25 % trypsin solution containing 0.02 % EDTA (Sigma-Aldrich).

Prior to each experiment, the cells were grown with the culture medium for a minimum of 24 h and then incubated at culture conditions for 24 h with BZP solutions prepared in fresh medium. *N*-benzylpiperazine dihydrochloride (purity ≥ 95 %, GC-FID, QNMR, Karl-Fisher) was purchased from LGS Standards Company (Australian Government National Measurement Institute). The stock solutions of the drug were prepared in methanol (HPLC Gradient Grade, LiChrosolv) and stored in a refrigerator (4 °C). The working drug solutions were prepared by an appropriate dilution of the stock drug solutions with culture medium. Cells growing in the same conditions in the medium with the addition of BZP solvent were utilized as a control.

### LDH Measurement

The selection of benzylpiperazine concentrations used for the study was determined by the measurement of the lactic dehydrogenase (LDH) in the medium after 24-h incubation of cells with BZP at the concentration range of 0.1, 0.3, 1, 3, 10, 30, 100, 300, and 1000 µg/mL (0.57 µM to 5.7 mM; three independent experiments in a few days apart) in accordance with the manufacturer’s protocol (CytoTox 96 Non-Radioactive Cytotoxicity Assay, Promega). The measurements were carried out by a Multiscan RC spectrophotometric microplate reader (Thermo/Labsystems) at 490 nm.

### ATP Generation Measurement

The intracellular ATP level was measured using the ATPlite™ Luminescence ATP Detection Assay System (Perkin Elmer). The control as well as cells cultured with BZP were detached and homogenized with Cell Lysis Solution from the reagent kit. The ATP concentration-dependent luminescent reaction of the luciferase/D-luciferin transformation was monitored by a GENios Reader (TECAN). The measurements were carried out in accordance with the manufacturer’s protocol. The concentrations of BZP: 57, 170; 570; and 1700 µM (10, 30, 100, and 300 µg/mL) were used in four independent experiments. The results were recalculated for the cellular protein content measured by the Lowry-Peterson method (Lowry et al. 1951) (Sigma-Aldrich).

### Monitoring of the Mitochondrial Membrane Potential (Δ*ψ*_m_)

Changes in the mitochondrial membrane potential (Δ*ψ*_m_) in the cells were monitored utilizing JC-1 cationic dye. After 24 h incubation with BZP, the cells were exposed to 2 mM JC-1 dye solution (MitoProbe Assay Kit, Invitrogen) and incubated for 45 min at 37 °C in darkness, then washed twice with PBS without Ca^2+^ and Mg^2+^ (Thermo Scientific), trypsynized with trypsin solution, and diluted in 2 mL of PBS. To confirm the sensitivity of the method as an additional positive control the mitochondrial uncoupler, carbonyl cyanide 3-chlorophenylhydrazone CCCP (50 µM, incubation for 45 min), was used. The concentrations of BZP: 57, 170, 570, and 1700 µM (10, 30, 100, and 300 µg/mL) were used in five independent experiments. Samples were analyzed by a BD FACSCanto II flow cytometer (Becton–Dickinson) using 488 nm excitation with 530/30 nm and 585/42 nm emission filters.

### ROS Production Analyzing

After incubation with the BZP, cells were loaded with 5 µM dichlorodihydrofluorescein diacetate (DCFH-DA) dye solution (Sigma-Aldrich) and incubated for 30 min at 37 °C in darkness. As a positive control method, a solution of H_2_O_2_ at a concentration of 1 mM was utilized in PBS. The dye-loaded cells were washed with PBS and treated the same way as during the Δ*ψ*_m_ measurement. The concentrations of BZP: 57, 170, 570, and 1700 µM (10, 30, 100, and 300 µg/mL) were used in seven independent experiments. The changes in ROS production were monitored by the BD FACSCanto II flow cytometer (Becton–Dickinson) using 488 nm excitation with a 530/30 nm emission filter.

### Determination of 8-OHdG

The OxiSelect™ Oxidative DNA Damage ELISA Kit (Cell Biolabs) assay was used for quantitative measurement of the oxidative DNA damage marker of the culture with BZP cells. Deoxyguanosine (dG) is one of the constituents of DNA and when it is oxidized it is altered to 8-hydroxy-2′-deoxyguanosine (8-OHdG). The concentrations of BZP: 570 and 1700 µM (100 and 300 µg/mL) were used in three independent experiments 8-OHdG measurement. The measurements and sample preparation were carried out in accordance with the manufacturer’s protocol. The protease K and Nuclease P1 were purchased in Sigma-Aldrich, and for the DNA isolation a QIamp DNA Mini Kit from Qiagen was used. The quality and quantity of DNA were confirmed by a NanoDrop ND-1000 spectrophotometry analysis. Absorbance of the ELISA product of reaction based on the measurements were read by the absorbance plate reader (GENios TECAN Reader) using 450 nm as the primary wave.

### Measurement of Caspases (-3, -9, and -8) Activity

Changes in the enzymatic activity of caspases: -3, -9, and -8 were analyzed utilizing Caspase Activity Assay Kits, Fluorimetric (Calbiochem), and DTT from Sigma-Aldrich. For detection of caspases’ activity, the LEHD substrate labeled with a fluorescent molecule, 7-amino-4-trifluoromethyl coumarin (AFC), was used. The method was based on the reaction monitored by a blue to green shift in fluorescence upon cleavage of the AFC fluorophore. The concentrations of BZP: 57, 170, 570, and 1700 µM (10, 30, 100, and 300 µg/mL) were used in three independent experiments for each caspase activity measurement. Fluorescence was measured using a fluorescence plate reader (LS 55, Perkin Elmer) utilizing an excitation of 390 nm and emission of 510 nm.

### Real-Time PCR Gene Expression Analysis

The selected genes for the gene expression analysis are presented in Table 1. Isolation of the cellular total RNA was performed with the GeneMATRIX Universal RNS/miRNA kit. Purification KIT (EURx) and TRIzol Reagent (Ambion by LifeTechnologies). The quality and quantity of the RNA were confirmed by analysis with the NanoDrop ND-1000 spectrophotometer. For the cDNA synthesis, the samples of 10 ng/µL of isolated RNA were used and the reverse transcription was performed in accordance to the manufacturer’s guidelines with the High Capacity cDNA RT kit with random primers (LifeTechnologies). The samples of cDNA at a concentration of 200 ng/mL were subjected to Real-Time PCR analysis with the TaqMan Gene Expression Master Mix (Life Technologies) and 7900HT Fast Real-Time PCR System. The concentrations of BZP: 57, 170, 570, and 1700 µM (10, 30, 100, and 300 µg/mL) were used in three independent experiments. The relative rate of expression calculated as the normalized C_T_ difference between the sample and control probe with adjustments for the amplification efficiency relative to the expression level of the housekeeping gene 18S RNA were calculated in accordance to the mathematical model of Pfaffl (Pfaffl 2001; Pfaffl et al. 2002).

**Table 1:** Genes analyzed during the studies

Gene	Name	Involved in the regulation of process
*BCL2*	B cell CLL/lymphoma 2	Apoptosis
*BAX*	BCL2-associated X protein	Apoptosis
*NFκB1*	Nuclear factor of kappa light polypeptide gene enhancer in B-cells 1	Apoptosis, inflammation
*RELA*	v-rel avian reticuloendotheliosis viral oncogene homolog A	Apoptosis, inflammation
*HSPA5*	Heat shock 70 kDa protein 5 (glucose-regulated protein, 78 kDa)	ER stress
*DDIT3*	DNA-damage-inducible transcript 3	ER stress
*SOD2*	Superoxide dismutase 2, mitochondrial	Removal of free radicals
*GPX3*	Glutathione peroxidase 3	Removal of free radicals
*CASP8*	Caspase 8, apoptosis-related cysteine peptidase	Apoptosis
*CASP9*	Caspase 9, apoptosis-related cysteine peptidase	Apoptosis

### Statistical Analysis

The results of individual experiments in which cells were treated with benzylpiperazine were calculated in relation to the results obtained for control cells (which were considered as 100 %). This way of presenting results was chosen in order to eliminate the influence of factors other than the compound BZP on the functions of cells. The results have been shown in the diagrams along with the designated values of standard errors of the mean (SEM) or standard deviations for the Real-Time PCR gene expression analysis where the results have been calculated in accordance to the mathematical model of Pfaffl (Pfaffl 2001; Pfaffl et al. 2002). Statistical analysis of the results was performed by the Statistica10 StatSoft progamme. The analysis was based on the system of random blocks and a linear overparameterized model treating the number of repetitions of the experiment between days (cells from another passage) as a random factor. In case of statistically significant differences (the test statistics F and the level of *p*), a post hoc analysis based on the Dunnett’s test was performed (the p level has been marked on the graph using the symbols: *p < 0.05, **p < 0.01; ***p < 0.001). Statistically significant differences were accepted for these results whereby the value of p was < 0.05.

## Results and Discussion

**Fig. 1:**
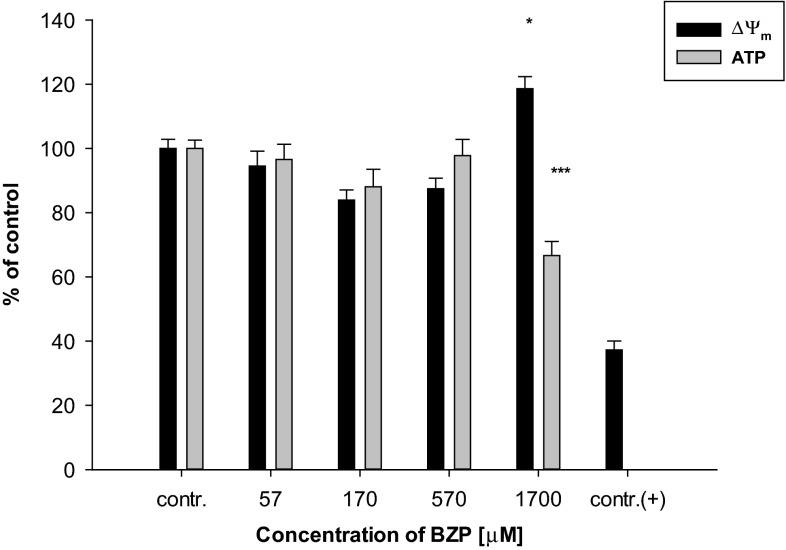
The influence of the selected concentrations of BZP on: the changes in potential (Δ*Ψ*_m_) of the inner mitochondrial membrane (*n* = 10–14; contr. (+): CCCP; system of random blocks: *F*(4, 42) = 5.7, *p* < 0.001; Dunnett’s test versus contr.: *p < 0.05); on the changes in ATP generation (*n* = 8; *F*(4, 28) = 10.4, *p* < 0.001; Dunnett’s test versus contr.: ***p < 0.001) in cell lines LN-18

The selection of benzylpiperazine concentrations utilized in the experiments was based on a method for evaluating the cytotoxicity of measuring the LDH released into the cells’ culture medium (the results have been shown as a percentage of the control). For the preliminary tests, a wide range of xenobiotic concentrations: 0.1, 0.3, 1, 3, 10, 30, 100, 300, and 1000 µg/mL, were used. Literature data reports indicate that in the case of a single dose of 200 mg of BZP, the content of this drug was estimated as 0.262 µg/mL in blood (Antia et al. 2009), which corresponds with preselected lower limit concentrations of the psychoactive substance. On the other hand, in the urine of poison victims a concentration of BZP as high as 202.7 µg/mL (Elliott 2011) was found, this is close to the upper range of concentrations selected for testing. Statistical analysis of the results has shown significant changes relative to the control in the release of LDH from tested cells under the influence of BZP at the two highest concentrations: 1700 µM (300 µg/mL) (110.7 % ± 9.0 %, *p* < 0.05) and 5700 µM (1000 µg/mL) (154.2 ± 14.3 %, *p* < 0.001). Since the purpose of this study was to determine the effect of BZP in inducing programed cell death parameters associated with oxidative stress and changes in energy state of cells, it was decided to reject the highest concentration as it was deemed too toxic, while for further experiments four levels of BZP were selected from a test range of 57, 170, 570, and 1700 µM (10, 30, 100, and 300 µg/mL). The highest concentration of the new psychoactive substance selected for analysis was similar to that used in experiments in the in vitro model of mouse heart muscle cells conducted by another research team (Arbo et al. 2014). The results presented in this paper indicate also that the chosen concentrations of BZP affected the inner mitochondrial membrane potential in the glial cell line LN-18 (Fig. 1).

Statistical analysis of the results has shown that culturing the cells in a medium containing the highest utilizing concentration of BZP has resulted in a statistically significant increase of Δ*ψ*_m_ relative to the control cells (118.6 ± 10.6 %, *p* < 0.05). In contrast, lower concentrations of BZP did not significantly affect the disruptions of the inner mitochondrial membrane proton gradient in the analyzed cells. Mitochondrial function can be disturbed after exposing the living cells to many toxic agents and those that are potentially toxic, such as ethanol (Manzo-Avalos and Saavedra-Molina 2010), a high-fat diet (Yu et al. 2014). and tobacco smoke (Yang et al. 2007), certain drugs and xenobiotics like cocaine, amphetamines, or compounds with a similar mechanism and type of action to BZP (Brown and Yamamoto 2003; Cunha-Oliveira et al. 2006).

**Fig. 2:**
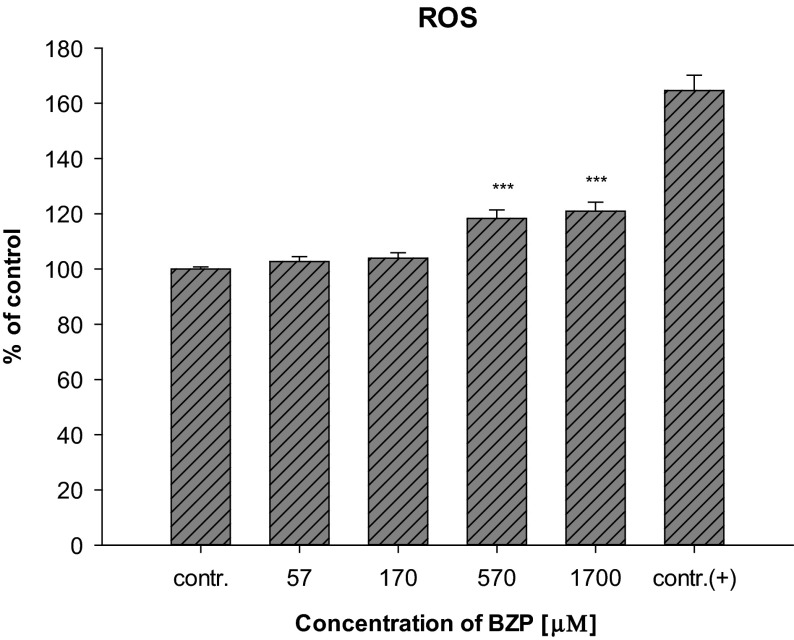
Changes in ROS production in cells of the LN-18 line under the influence of BZP (*n* = 14; contr. (+) 1 mmol/L H_2_O_2_; system of randomized blocks: *F*(4, 52) = 18.1, *p* < 0.001; Dunnett’s test versus contr.: ***p < 0.001)

Mitochondria, as organelles involved in free radical generation as well as in ATP synthesis, play an important role in programed cell death (Wang and Youle 2009). Dysfunction of those organelles by the influence of proapoptotic factors may result in changes in the electrical potential of the inner mitochondrial membrane (Δ*ψ*_m_) due to abnormalities in the formation of a proton gradient across the membrane. Changes Δ*ψ*_m_ not only result in impaired production of ATP in the cells, but may also contribute to an increase in the permeability of the mitochondrial membrane megachannels and, consequently, the activation of a caspase cascade (Marzo et al. 1998). A statistically significant decrease of the ATP level relative to the control level (66.6 ± 12.4 %, *p* < 0.001) in cells from the line LN-18 grown in the medium with the presence of BZP at a concentration of 1700 µM (300 µg/mL) has been shown (Fig. 1). The changes in the mitochondrial membrane potential observed in LN-18 cells caused by the action of BZP may cause excessive formation of ROS, leading both to the oxidization of molecules including lipids, proteins, or DNA, as well as disorders of electron transport through the mitochondrial membrane resulting in lowering of ATP levels in the cells (Dimroth et al. 2000). Reactive oxygen species are byproducts of oxidative phosphorylation occuring through the mitochondrial electron transport (Murphy 2009). Indeed, BZP increased the production of reactive oxygen species in the LN-18 cells (Fig. 2).

Statistically significant differences were observed at the two highest concentrations of the BZP: 570 µM (100 µg/mL) (118.3 ± 11.6 %, *p* < 0.001) and 1700 µM (300 µg/mL) (120.9 ± 12.3 %; *p* < 0.001), suggesting a correlation between the observed changes in the potential of the inner mitochondrial membrane and the depletion of the ATP content in glial cells.

The changes observed in the cells indicate that BZP has a significant impact on a change in the functioning of the mitochondrial respiratory chain in the test cell line. The reported abnormal mitochondrial membrane potential may be a consequence arising from disturbances in the electron flow. Changes in Δ*ψ*_m_, generated by the action of cytotoxic agents, including increased levels of calcium ions released from the endoplasmic reticulum, may be accompanied by the opening of the mitochondrial megachannels and the release of proapoptotic factors from the intermembrane space into cytoplasm, activation of which is introduced to the cell on the path of death (Łabędzka et al. 2006). The similar influence of piperazine derivatives leading to the disturbances in Ca^2+^ homeostasis was observed during the studies of Arbo et al. (2015). The disruption of Δ*ψ*_m_ in cells for line LN-18 treated with BZP was accompanied by a decrease of intracellular ATP as well as a corresponding increase in production of reactive oxygen species. The relationship between the mitochondrial membrane potential, the level of ATP, and/or generation of reactive oxygen species have been the subject of research conducted for both in vitro and in vivo studies using a variety of experimental models (Klingenberg and Rottenberg 1977; Wu et al. 1990; Waterhouse et al. 2001; Ricci et al. 2003). Analysis of the experiments described in the literature indicate that the observed disruption of the oxidative phosphorylation process is manifested by a decrease in the mitochondrial membrane potential and is associated with a concomitant decrease of the intracellular concentration of ATP and an enhanced production of reactive oxygen species (and nitrogen). The results of the experiments performed on a murine heart, despite the disruption of the mitochondrial membrane potential revealed in evidence, observed reduction in ATP levels in the cells after incubation with BZP at concentrations similar to those used in this paper (Arbo et al. 2014). The confirmation of this thesis could be also observations made by the team of Arbo et al. (2015), who has studied the impact of BZP among other piperazine derivatives on the differentiated human neuroblastoma SH-SY5Y cells. The team observed the impact of BZP that the increase in intracellular Ca^2+^ levels leads to mitochondrial hyperpolarization and to the conservation of ATP levels. In summary, the results of the experiments presented in this paper as well as and as of the research conducted on H9c2 rat cardiomyoblasts (Arbo et al. 2014) and human neuroblastoma SH-SY5Y cells (Arbo et al. 2015) have shown that disparities in the effects of the cytotoxic function of mitochondria may be the result of not only the differences in the type of compound and its concentration, but also the cellular model.

The correlation between the changes observed and presented here in the inner mitochondrial membrane potential and production of reactive oxygen species were also described by other authors (Suski et al. 2012). They demonstrated that the increased production of ROS may be not only an uncoupling effect of the mitochondrial respiratory chain but also may result from the high mitochondrial membrane potential that was observed in cell lines LN-18 cultured with BZP at the highest concentrations. The reason for the increased production of ROS accompanying increased Δ*ψ*_m_ in glial cells LN-18, with a simultaneous decrease of the intracellular concentration of ATP due to the new psychoactive substance’s influence, might be inhibition of the mitochondrial ATP synthase because of the influx of calcium from the endoplasmic reticulum to the mitochondrial matrix. This mechanism has been observed as an element of the so-called “Crabtree effect” in cells, especially cancer, having high proliferative potential, wherein the process of glycolysis is mainly involved in the production of ATP (The Warburg effect). Studies on tumor cells Zajdel and Ehrlih (Wojtczak et al. 1999; Marroquin et al. 2007; Diaz-Ruiz et al. 2011) have shown that increased levels of Ca^2+^ in the cytoplasm by the influence of glucose results in an increased concentration of calcium ions in mitochondria. These ions cause inhibition of ATP synthase complexes through interaction with the F_1_-F_0_ enzyme, thereby reducing the production of ATP, increasing the potential of the inner mitochondrial membrane and increasing the amount of reduced forms of mitochondrial nicotinamide dinucleotides. These two latter phenomena are explained as a result of competition occurring between glycolysis and oxidative phosphorylation (Wojtczak et al. 1999). Since the line LN-18 is a tumor cell line, these effects may explain the results of the conducted experiments regarding the relationship between the increased Δ*ψ*_m_ and decreased ATP level.

The role of ROS in the mechanism of neurodegeneration type of action has been explained with reference to compounds with a similar mechanism of action and psychostimulant properties like BZP (Brown and Yamamoto 2003; Montiel-Duarte et al. 2004; Cerretani et al. 2011). These results suggest that excessive production of ROS may be involved in hyperthermia, which is one of the effects of action of amphetamines. Hyperthermia leads to the fact that energy instead of storage of the high-bond ATP is dissipated as heat which is then accompanied by increased production of ROS (Brown and Yamamoto 2003; da Silva et al. 2014). Since hyperthermia is listed among the adverse effects of BZP (Gee et al. 2008; Schep et al. 2011), it can be speculated that it is evidence of impaired function of the mitochondrial electron chain and oxidative stress in cells intensified under the influence of the new psychoactive substance. For other xenobiotics, e.g., MDMA, the role of the metabolites such as quinones and thioesters in the formation of ROS and oxidative stress have been indicated (Moon et al. 2008; Song et al. 2010).

**Fig. 3:**
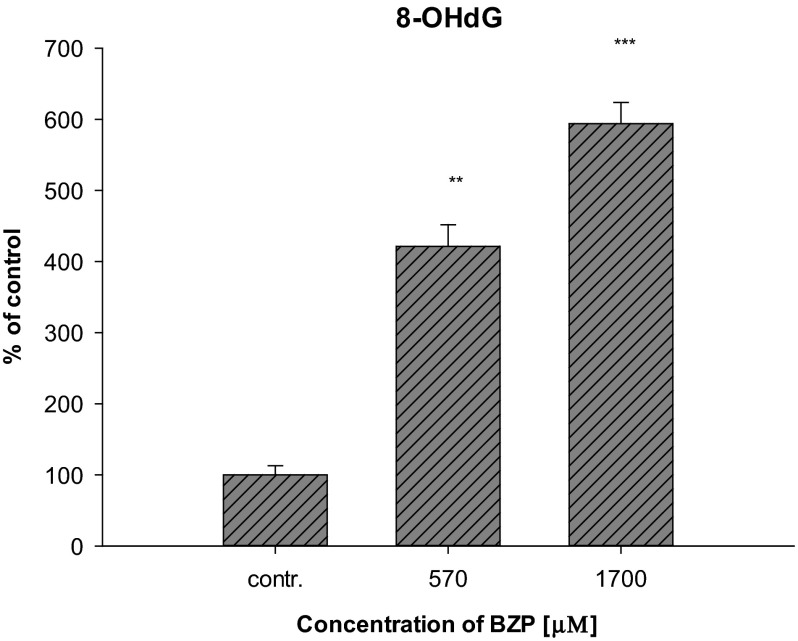
Influence of BZP on changes in the concentration of 8-OHdG in cell lines LN-18 (*n* = 3; system of randomized blocks: *F*(2, 4) = 99.7, *p* < 0.001; Dunnett’s test versus contr.: **p < 0.01; ***p < 0.001)

The increased production of reactive oxygen species in LN-18 cells under the influence of BZP reported after the experiments may also suggest intensification of oxidative stress. ROS are products of metabolism occurring in the cells—in a low level, physiologically they play the main role in intracellular signaling and the regulation of biochemical processes, but their over-production and depletion of antioxidant mechanisms leads to the development of oxidative stress in cells. Many pathological states and diseases are associated with severe oxidative stress. Impaired intracellular redox homeostasis can lead to irreversible changes in the chemical structure of molecules resulting from the oxidation of proteins, lipids, and DNA (Circu and Aw 2010). Since BZP affected the formation of ROS in glial cells cultured in a medium containing BZP at concentrations of 570 and 1700 µM (100 and 300 µg/mL), an experiment aimed at determining whether the xenobiotic in these concentrations significantly affects the formation of oxidative DNA damage has been performed. A marker of such damage, 8-hydroxy-2′-deoxyguanosine (8-OHdG), was analyzed in cells from the line LN-18. The results have shown (Fig. 3) that in the case of the concentration of 570 µM (100 µg/mL) and 1700 µM (300 µg/mL) the level of the DNA damage marker statistically increased to: 421.4 ± 52.7 % (*p* < 0.01) and 593.8 ± 51.5 % (*p* < 0.001), respectively, confirming the effect of BZP on the reinforcement of the activation of oxidative stress.

**Fig. 4:**
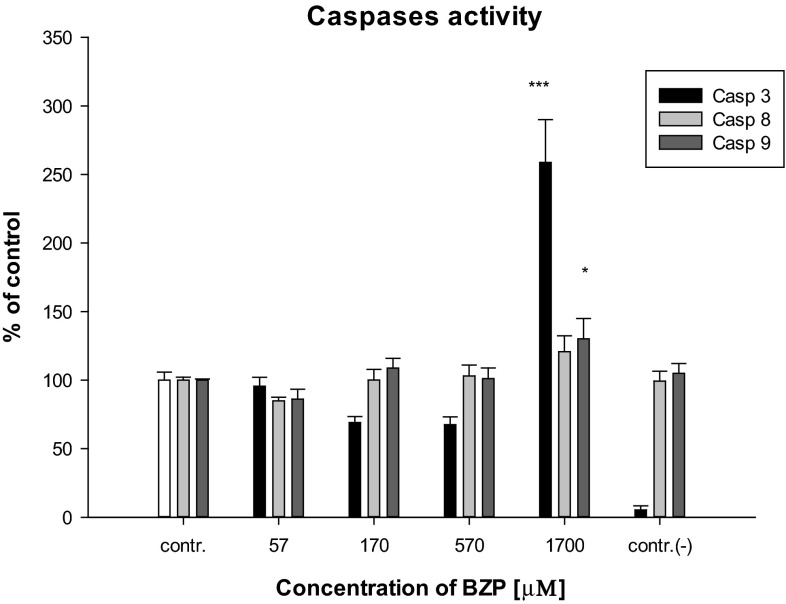
Influence of BZP on: caspase-3 activity (*n* = 5–6; system of random blocks: *F*(4, 22) = 27.6; *p* < 0.001; Dunnett’s test versus contr.: ***p < 0.001), caspase-9 activity (*n* = 5–6; *F*(4, 22) = 27.4; *p* < 0.001; Dunnett’s test versus contr.: *p < 0.05), caspase-8 activity (*n* = 5–6; *F*(4, 22) = 2.3; *p* < 0.05 in cell lines LN-18; contr.(−) cells were cultured in the presence of BZP at concentration of 300 µg/mL with the addition of the inhibitor

In order to determine the impact of BZP on apoptosis of cells, the activation of the executive caspase, caspase-3, was analyzed. Changes in the activity of caspase-3, depending on the concentration of BZP in the cultured glial cells, are shown in Fig. 4.

The results have shown that BZP in the lowest concentration used caused a statistically significant increase of caspase-3 activity at the level of 258.8 ± 76.4 % (*p* < 0.001) relative to the control, which indicates that apoptosis has begun in LN-18 cells after treatment with the drug. The observed influence of xenobiotics on activation of the proteolytic enzyme may suggest that BZP is the factor inducing programed cell death. A similar effect on the activation of caspase-3 has been shown for amphetamine, a compound with comparable effects of action to BZP in liver cells (Montiel-Duarte et al. 2002; Cunha-Oliveira et al. 2006). Due to the increased caspase-3 activity under the influence of the highest of the tested concentrations of BZP, attempts to determine the induction of the programed cell death pathway by analyzing the activities of the two initiating apoptosis caspases, caspase-8 (related to the receptor pathway), and caspase-9 (bound to the mitochondrial pathway), were made. Analysis of the results, shown in Fig. 4, revealed that BZP used in the highest concentration of 1700 µM (300 µg/mL) caused the activation of caspase-9 (130.2 ± 36.3 %, *p* < 0.05). An increased activation of caspase-9 in cell lines LN-18 treated with xenobiotic indicates that BZP may influence the induction of the mitochondrial pathway of apoptosis in glial cells. Activation of the caspase is related to the formation of apoptosome and release of cytochrome c, apoptosis-inducing factor (AIF) or HtrA serine peptidase 2 (HTRA2) protein and calcium ions from the mitochondria of cells following the opening of the mitochondrial megachannels (Łabędzka et al. 2006). The observed increase Δ*ψ*_m_ and increased production of ROS in cell lines LN-18 treated with BZP suggests that these phenomena lead in turn to activation of caspase-9 with consequent cell death. Similar mechanisms are postulated for the neurodegeneration properties of amphetamine when tested on neuronal cells on the frontal cortex of rats during in vitro experiments (Cunha-Oliveira et al. 2006). On the other hand, as a result of the statistical analysis of the data it was found that BZP does not significantly affect changes in caspase-8 activity when compared to control cells (Fig. 4). The results indicate that there is no basis to conclude that BZP induces apoptosis in LN-18 via the receptor pathway, as was demonstrated for amphetamine and cocaine in neuronal cells (Cunha-Oliveira et al. 2006) and opposite to MDMA, whose affinity for TNF-α in rat liver cells was confirmed during in vivo experiments (Cerretani et al. 2011). A low affinity of BZP to receptor cell membranes may also be a result of the lipophilic properties of this compound (octanol/water for BZP logP = 1.3; for MDMA logP = 2.2—according to the PubChem database), which are weaker than MDMA.

**Fig. 5:**
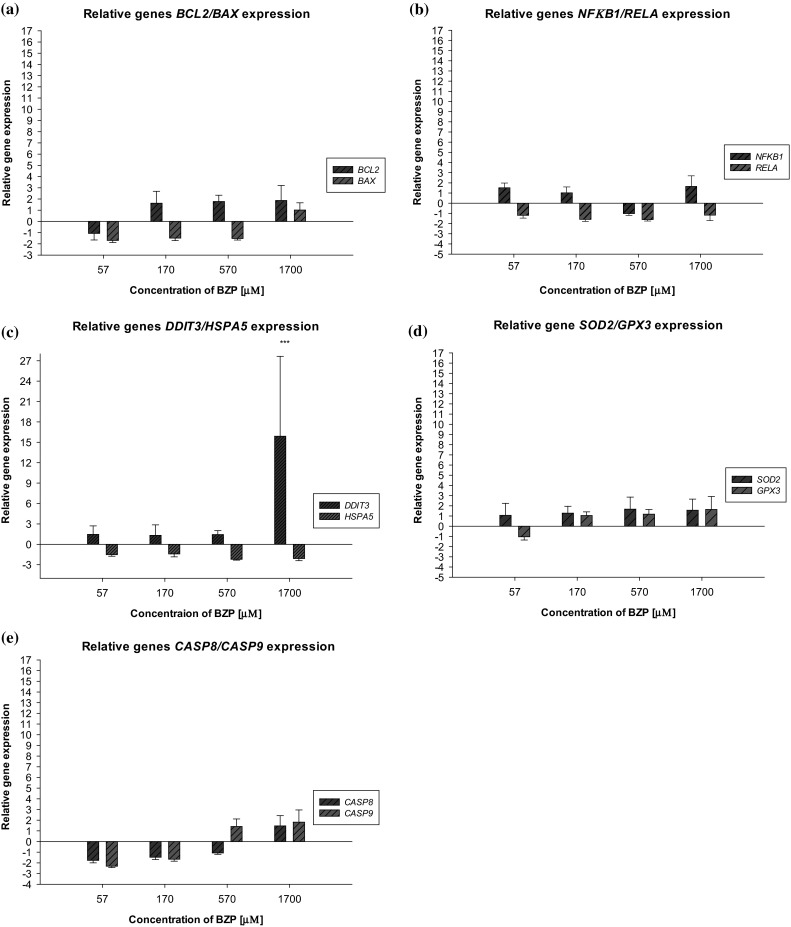
Influence of BZP on the changes in the level of relative gene expression. **a** *BCL2/BAX*; **b** *NFκB1/RELA*, **c** *DDIT3/HSPA5*, **d** *SOD2/GPX3*, and **e** *CASP8/CASP9* in the cells of the LN-18 line (*n* = 3–4, pair-wise fixed randomisation Test© versus contr. *p < 0.05; ***p ≤ 0.001)

In order to complete the understanding of the influence of BZP on glial cells, analysis of the changes in the relative expression of selected genes associated with apoptosis and the trail induction of this process, severe oxidative stress in cells, mitochondrial dysfunction, and shocking the endoplasmic reticulum has been performed. In Fig. 5, the relative expression levels of genes encoding proteins: BCL2, BAX, NFB1, RELA, DDIT3, HSPA5, CASP8, CASP9, SOD2, and GPX3 in the cells of the LN-18 line after incubation with BZP were presented. BZP used at a concentration of 57 and 170 µM (10 and 30 µg/mL) resulted in a decline of the relative gene expression of *CASP9*. For a concentration of 57 µM (10 µg/mL), the relative gene expression was −2.3 ± 0.1 (*p* < 0.05), while for a concentration of 170 µM (30 µg/mL): −1.66 ± 0.2 (*p* < 0.05).

There have been some cases known where xenobiotics at low concentrations protect the body’s cells (Shatrov and Brüne 2003; Lechanteur et al. 2005; Wätjen et al. 2005), but in the studies described in this paper such action of benzylpiperazine can only be speculated and this is supported by the results of other experiments. Additionally, an increase of the relative expression of the *DDIT3* gene to 15.9 ± 11.8 (*p* = 0.001) compared to the control (the level gene expression in control cells is defined as 1) in glial cells under the effect of the legal high in the highest tested concentration was observed. Increased gene *DDIT3* expression in cell lines LN-18, resulting from usage of the highest concentrations of BZP, may suggest activation of the ER shock by the influence of a toxic substance. Signals’ death being the effect of inducing shock in humans’ ER may be attributed to mitochondria, whose disruption has been demonstrated during the conducted experiments.

The results of the experiments lead to the conclusions that BZP affects largely on the mitochondrial dysfunction and associated with that disturbances of energy state of the cells. Moreover, the results of Real-Time PCR gene expression analysis (increased gene *DDIT3* expression) indicate that disruption of cellular function is connected with the disorders of the endoplasmic reticulum possibly associated with homeostasis of calcium ions which may be supported by the findings of other authors (Arbo et al. 2015).

Phsychostimulating properties of BZP as well as its impact on the central nervous system have been subjected to a wide range of scientific research for many years. Experiments in animals suggest that BZP has direct and indirect sympathomimetic activities, stimulates the release and inhibits reuptake of dopamine (DA), serotonin (5-HT), and noradrenaline (NA) (Campbell et al. 1973; Magyar et al. 1986; Szücks et al. 1987), however, the impact on noradrenergic and serotonergic activity is not entirely clear and to the present day being considered (Simmler et al. 2014). It also shows the positive impact of BZP on the NA level observed in the hypothalamus, the DA in the striatum, and 5-HT in the hippocampus of rats exposed to long-term the psychoactive substance (Tekes et al. 1987). In vitro studies carried out on rat synaptosomes by a group of researchers led by Baumann et al. (2005) in order to compare the mechanism of action of BZP, TFMPP, and MDMA have demonstrated that BZP causes the release of the organic cations ions [3H]MPP^+^ in dopamine transporter (DA). These ions, as the neurotoxins, may not exceed the blood–brain barrier, but can lead to the death of dopaminergic neurons in the substantia nigra, which may indicate to the neurotoxic effect of BZP.

Described in this paper, results of the research on the effects of BZP directly on the cells of the nervous system may be additional information about the mechanism of psychostimulating action of BZP and its postulating toxic impact on the nervous system.

## Conclusions

The research presented in this paper is a modern approach to the issue of the mechanism of action of xenobiotics at the cellular level. The experiments conducted in order to demonstrate the mechanism of toxicity of new psychoactive substances on the body seem to be relevant for the model assessment of the possible dysfunction of cells arising from the use of the selected model drug from the new psychoactive substances—BZP. Analysis of the parameters associated with the impaired mitochondrial function carried out and described in this paper, such as changes in the potential of the inner mitochondrial membrane Δ*ψ*_m_, ATP production, and generation of ROS, made it possible to assess the impact of BZP both on the mitochondrial function of glial cells LN-18, inducing the process of programed cell death as well as the energy state and intensification of oxidative stress of the tested type of cells. The results presented in this paper have shown that the toxicity of BZP cells refers to disorders of mitochondrial function leading to increased ROS production, disorders of the biosynthesis of ATP, free radical damage to the cell endogenous compounds’ structure (including DNA), and induction of apoptosis. On this basis, it can be concluded that the toxic effects of the cells cultured with BZP are exposed especially on those organs whose cells have a large number of mitochondria, such as the neuronal cells.
